# The Clearing Hospitals for the Territorial Medical Service

**Published:** 1912-07-13

**Authors:** 


### THE ROYAL ARMY MEDICAL CORPS SECTION.

### The Clearing Hospitals for the Territorial Medical Service.

In the number of The Hospital for September 29, 1909, we outlined the position and function of the Clearing Hospital for the Regular Army in the system of aid to the sick and wounded in war, and showed what an important part it played in the organisation for the removal of the casualties in war and their conveyance to a suitable place for the necessary treatment. It was there explained that the clearing hospital is the first unit of the Medical Service in the evacuating zone, and is, in fact, really "the pivot upon which the whole system of evacuating sick and wounded turns," and that it is officially described as a mobile unit which receives the sick and wounded from the field ambulances and transfers them, by various methods, to the stationary hospitals and to the ambulance trains. A clearing hospital should be able to accommodate 200 patients and is staffed in the same proportion as a stationary hospital, so that its personnel consists of eight medical officers, one quartermaster, and eighty rank and file. No Army nurses serve with it. In the previous article to which we are referring it was indicated that at that time the clearing hospital had no existence in the Territorial Medical Service, but that there were indications that the military authorities proposed to enlist the services of the voluntary aid organisations existing in the country for furnishing it. This idea has now been carried out, and it has been decided that in the event of war in the home territory the provision of these necessary clearing hospitals is one of the most important of the duties assigned to the voluntary aid detachments, who will also find the necessary personnel for them.

This duty of improvising hospitals—whether clearing ones to receive sick and wounded from the field ambulances or temporary ones to take over the patients evacuated by the clearing ones—is one requiring special skill, inasmuch as buildings may sometimes have to be suitably converted for the reception of wounded, or, if buildings are not available, then extemporised shelters may have to be erected to receive them. To convert buildings, such as country or farm houses, public buildings, or, in fact, whole villages or small towns into temporary hospitals for the care and shelter of sick and wounded until they can be placed in railway trains for conveyance to general hospitals, calls for a knowledge of the necessary cubic space required for each patient, of the principles of ventilation, of the need for a proper provision of light and sanitary facilities, and of the preparation of rooms and wards, while the furnishing of these latter must also tax the ingenuity of the detachments, for in many villages the supply of bedsteads, mattresses, and bedding will be very limited and recourse will have to be had to improvised beds, mattresses, and pillows. Again, when buildings are not available and resort must be had to extemporising tents and shelter for sick and wounded, a good deal of skill will be called for and many devices will have to be resorted to so as to obtain quickly the required accommodation. In view of this, special instruction is given to the men's detachments in this branch of the work and it is made a prominent feature in their training, especially in their voluntary aid displays, so that the detachments very generally understand that to make a building suitable for use as a temporary hospital it must have large rooms, broad doorways, facilities for ventilation, be furnished with sanitary arrangements, and have a good supply of water, while additional recommendations are its proximity to the railway station, open ground around it, and the presence of buildings near it that might be utilised for stores.

Valuable, however, as all such training is in time of peace, it becomes of no practical use in time of war unless it is applied systematically and methodically and under proper direction. The possibility of things not working smoothly in the confusion of hostilities is favoured by the organisation of the voluntary aid detachments, seeing that they are all independent bodies with their own commandants, and consequently inclined to act on their own account. This has now been recognised by those in authority, and we understand that the question has been under discussion at a recent meeting of the War Office Advisory Committee on the voluntary aid detachments. After a full consideration of the matter we learn that it was decided that there was great risk under present arrangements of confusion arising on the arrival of a number of independent detachments in a district where hostilities were imminent or were actually in progress, unless there was some recognised authority to whom they could report themselves and under whose orders they would work. Accordingly a recommendation has gone forward from the Advisory Committee to the military authorities that there should be sanctioned for each division of the Territorial Force the cadre of a clearing hospital, to consist of one lieutenant-colonel, one captain, one quartermaster, one sergeant, one corporal, and three privates, giving a total of eight for each division, or a personnel of 112 for the fourteen divisions. These cadres would belong to the Territorial Medical Service and their training would consist in making them conversant with all the duties which would devolve upon a clearing hospital of the Expeditionary Force and as laid down in Royal Army Medical Corps training. They would probably be borne as supernumerary on the strength of one of the field ambulances and would get their training at one of the schools of instruction. In war the cadres would mobilise with their divisions and would take charge of and superintend the work of the voluntary aid personnel. Such an arrangement differs from that at present in vogue. The existing plan as laid down in the official schemes for the organisation of voluntary aid in England and Wales and also in Scotland is that a retired officer (not liable to recall) of any

Regular arm of the Service, but preferably a retired medical officer, is to be nominated by the general officer commanding each Territorial division, and that his duties would be to superintend, under the Army medical authorities on the lines of communication, the arrangements for the evacuation and care of the sick and wounded as far as the voluntary aid detachments are concerned in this work.

It is to be hoped that the formation of this suggested clearing hospital cadre will be approved of by the military authorities and sanctioned by the Treasury, as it should materially assist in that methodical and harmonious working of the voluntary aid detachments which is so necessary for the proper discharge of their duties and thus strengthen what we have all along felt was a weak point in their organisation. In peace time, too, these cadres could also be of great help to the detachments, for they could train in conjunction with large bodies of voluntary aid personnel when giving any displays. Such displays would probably be best held in the neighbourhood of towns and large villages where the personnel could be accommodated, and at any time when military manœuvres are being carried out it might be arranged that a certain number of the troops should fall out as wounded and allow any local voluntary aid detachment to attend to them and provide them with shelter. It is not necessary, nor is it desirable, for voluntary aid detachments to go into camp to learn their work as there they would be working with regulation equipment and under conditions quite different from those they would find around them should they be called out in case of invasion. They will derive more benefit from "field-days" in their own neighbourhood, when they take out with them whatever equipment they may have in their possession and rely on supplying their needs by utilising local resources. Such displays also help to develop and popularise the voluntary aid movement and thus keep up enthusiasm in the work. Unfortunately such displays cost money and no financial assistance is to be looked for from Government, but so far a very generous public has provided the necessary means for the several excellent demonstrations that have already taken place, and we feel confident there will be a continuance of this liberality. For the present the voluntary aid detachments must continue their training without the help of the cadres, for as yet the latter are only suggested, and it often takes a considerable time before proposals of this kind materialise at the hands of the military authorities.

